# Criteria air pollutants and hospitalizations of a wide spectrum of cardiovascular diseases: A nationwide case-crossover study in China

**DOI:** 10.1016/j.eehl.2022.10.002

**Published:** 2022-11-17

**Authors:** Cong Liu, Renjie Chen, Xia Meng, Weidong Wang, Jian Lei, Yixiang Zhu, Lu Zhou, Haidong Kan, Jianwei Xuan

**Affiliations:** a School of Public Health, Shanghai Institute of Infectious Disease and Biosecurity, Key Lab of Public Health Safety of the Ministry of Education and NHC Key Lab of Health Technology Assessment, Fudan University, Shanghai 200032, China; b Children’s Hospital of Fudan University, National Center for Children’s Health, Shanghai 201102, China; c Shanghai Institute of Pollution Control and Ecological Security, Shanghai 200092, China; d Health Economic Research Institute, School of Pharmacy, Sun Yat-Shen University, Guangzhou 510275, China

**Keywords:** Criteria air pollutants, Hospitalization, Cardiovascular disease, Cause-specific, Case-crossover study

## Abstract

Few national studies have systemically examined the effects of criteria air pollutants on cardiovascular morbidity. This study aimed to investigate the associations between all criteria air pollutants and hospitalization of cause-specific cardiovascular diseases (CVD) in China. We obtained data on CVD hospitalization events of four major categories and 12 specific diseases from 153 hospitals distributed in 20 provincial-level regions from 2013 to 2020. We adopted a time-stratified case-crossover study design using individual cases to capture the effect of short-term exposure to six criteria air pollutants on CVD hospitalizations, using conditional logistic regression models. More than 1.1 million CVD hospitalization events were included. The lag pattern exploration demonstrated the largest effect for six air pollutants on lag 0–1 day. PM_2.5_, PM_10_, NO_2_, and CO were significantly associated with increased hospitalization from ischemic heart diseases, cerebrovascular diseases, other heart diseases, and five specific causes of CVD. The effect estimates of NO_2_ were the most robust when adjusting for co-pollutants. The concentration-response curves were positive and linear for most pollutant–endpoint pairs (except for O_3_), and these positive associations remained even below the 24-h levels recommended by WHO Air Quality Guidelines and China Air Quality Standards. This nationwide case-crossover study in China demonstrated that short-term exposure to multiple ambient air pollutants may significantly increase the risk of cause-specific CVD hospitalizations even under the most stringent air quality regulations, striking an alert for potential CVD patients against these environmental risk factors.

## Highlights

• A national hospital-based registry for a wide spectrum of CVD hospitalization.
• We evaluated the associations of short-term exposure to six criteria air pollutants.
• PM_2.5_, PM_10_, NO_2_, and CO were significantly associated with increased CVD hospitalization.
• Five specific causes of CVDs were closely linked with exposure to air pollution.
• We observed adverse effects of air pollution below the recent WHO Air Quality Guidelines.

## Graphical abstract

**Figure:**
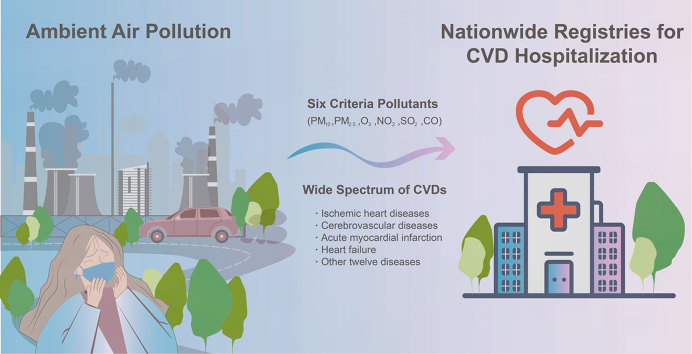

## 1 Introduction

Cardiovascular disease (CVD) is a group of physiological disorders in the circulation system, which mainly includes coronary heart disease, cerebrovascular disease, strokes, and other conditions [1]. CVD is the world’s leading cause of death, taking more than 17.9 million lives each year, accounting for 32% of all deaths worldwide [2]. CVD is also the greatest threat to the global disease burden. It was estimated to be the top-ranked causes of disability-adjusted life years (DALYs) for adults and the elderly by the 2019 Global Burden of Disease study (GBD 2019) [3]. Behavioral risk factors for CVD, such as an unhealthy diet, low physical activity, alcohol consumption, and tobacco use, can be measured in primary care facilities by personal intervention, but environmental risk factors of CVD require inter-governmental efforts.

Over the past two decades, a growing body of scientific literature has raised concern about the adverse health effects of environmental risk factors on CVD [[4], [5], [6], [7]]. Ambient air pollutants have been commonly reported to be associated with an increased risk of CVD mortality and morbidity [[8], [9], [10], [11], [12]]. These pollutants include particulate matter (PM) classified by aerodynamic diameters as inhalable particles (PM_10_) and fine particles (PM_2.5_), ozone (O_3_), nitrogen dioxide (NO_2_), carbon monoxide (CO), and sulfur dioxide (SO_2_). Although numerous epidemiological studies have separately assessed the cardiovascular impact of certain air pollutants at the city or regional level, few studies have systematically evaluated the multicity health effects of all criteria air pollutants on all specific causes of CVD, especially at the national level.

More than 75% of CVD deaths occurred in low- and middle-income countries (LMICs), but scientific evidence on CVD has been mostly gathered in developed regions [13]. China is the largest developing country and is facing severe air pollution problems that have recently attracted great research interest, but the results are generally mixed and incomparable [8,[14], [15], [16]]. For instance, Ren et al. [15] reported significant associations between PM_10_, NO_2_, O_3_, and CVD hospitalization in Shenyang. However, a similar study in Lanzhou found significant effects only for PM, but not for NO_2_ or O_3_ [16]. There is also a need to investigate the subclass of cardiovascular endpoints, since the majority of studies have only examined total CVD and were unable to identify specific CVDs that are more sensitive to air pollution. Therefore, we designed this nationwide case-crossover study in China to evaluate the associations of short-term exposure to six criteria air pollutants with the hospitalization of a wide spectrum of CVDs based on a nationwide hospital-based registry.

## 2 Materials and methods

### 2.1 Data collection

Daily hospitalization events were obtained from the SuValue database, details of which have been described elsewhere [17]. In brief, the SuValue database is a hospital-based registry across 20 provincial-level regions in China. It generates a structured patient-level database after extracting, validating, and aggregating medical records directly from the Hospital Information System. In this analysis, we extracted hospitalization data for a wide spectrum of CVDs from 153 hospitals in the SuValue database from 2013 to 2020 (Fig. S1). The primary diagnosis was coded according to the International Classification of Diseases, 10th version (ICD-10) [18]. Four major categories of CVD, which have been linked to air pollution in previous studies [6,12,14,19], were derived according to the coding database of the National Healthcare Security Administration, including ischemic heart diseases (IHD, I20–I25), pulmonary heart diseases (I26–I28), other forms of heart disease (I30–I52), and cerebrovascular diseases (I60–I69). Hospitalizations among these categories were further classified into specific causes of CVD corresponding to each ICD code, including angina pectoris (I20), acute myocardial infarction (I21), acute IHD (I24), chronic IHD (I25), other pulmonary heart diseases (I27), myocardiopathy (I42), paroxysmal tachycardia (I47), heart failure (I50), heart disease complications (I51), cerebral infarction (I63), occlusion/stenosis of cerebral artery (I65), and other cerebrovascular diseases (I67). Data on the date of admission, gender, and age of each patient were acquired, and information was linked with a unique and anonymized identifier. Data authorization was obtained from the SuValue database before the initiation of this analysis; thus, no ethical consideration or informed consent was needed. The study protocol was approved by the Institutional Review Board at the School of Public Health, Fudan University (IRB#2021-04-0889) with a waiver of informed consent.

### 2.2 Assessment of exposure

Due to the confidentiality agreement, exposure assessment was only practical at the hospital level. We geocoded the hospital addresses and assigned the exposure level of environmental factors based on the nearest fixed-site monitoring stations. Daily concentrations of six criteria air pollutants measured in the nearest air quality stations, including PM_10_, PM_2.5_, O_3_, NO_2_, SO_2_, and CO, were collected from the National Air Quality Monitoring System. The median distance between each hospital and monitoring station was 14 km (ranging from 0.4 to 25 km). Metrological factors, including daily mean temperature and mean relative humidity, were monitored at the nearest meteorological stations.

### 2.3 Statistical analysis

A time-stratified case-crossover study design was adopted to evaluate the association between short-term exposure to six criteria air pollutants and hospitalizations of CVDs [[20], [21], [22]]. The date of hospitalization event for each patient was defined, and the control days were selected as the days in the same month that shared the same day of the week [22,23]. There were usually three or four control days for each case day. Conditional logistic regression models were applied to investigate the associations. We matched each of the CVD hospitalization events of four major categories and 12 specific diseases with a daily concentration of each of the six air pollutants on case/control days. Due to the case-crossover study design, time-invariant covariates need not be adjusted as cases serve as their controls. We only adjusted for temperature (lag 0–3 days) and relative humidity (lag 0–3 days) according to previous studies [24,25], using natural spline functions with degrees of freedom (*df*) of 3. We explored potential lag patterns on single lag days from 0 to 3 and corresponding moving averages. The lag that generates the largest estimate with the best convergence was adopted as the main lag structure for subsequent analyses. Those observations with missing values on any covariates were excluded by the model. The exposure-response curves were pooled by introducing non-linear terms via natural splines with 3 *df*s. Two pollutant models were built by including each of the co-pollutant at one time to test the robustness of the associations. Since atmospheric pressure is associated with air pollution levels [26], and it has been reported to be a short-term predictor for CVD events [27], we conducted a sensitivity analysis by including atmospheric pressure (natural splines with 3 *df*s) as a covariate to test whether it has a confounding effect on the associations between air pollutants and CVD.

We performed the statistical analyses using R software (Version 3.6.1) with the “survival” package. The estimated associations were expressed as the percentage change in mortality, and its 95% confidence intervals (95%CI) were associated with a 10 μg/m^3^ increase in air pollutant concentrations, except for a 0.1 mg/m^3^ increase for CO. The statistical tests were two-sided, and *P* values < 0.05 were considered to be statistically significant. A false discovery rate was used to adjust *P* values for multiple corrections.

## 3 Results

This analysis included the number of hospitalization events for CVD (Table 1). For larger categories, there were 387,817 cases of hospitalization from IHD and 558,289 cases from cerebrovascular diseases during the study period. For specific diseases, chronic IHD (N = 346,684) and cerebral infarction (N = 339,629) accounted for the largest proportion.

**Table 1:** Summary of numbers of hospitalization of cardiovascular diseases.

Disease names	ICD codes	Counts
Ischemic heart diseases	I20–I25	387,817
Pulmonary heart/circulation diseases	I26–I28	26,594
Other forms of heart disease	I30–I52	153,088
Cerebrovascular diseases	I60–I69	558,289
Angina pectoris	I20	17,905
Acute myocardial infarction	I21	15,416
Other acute ischemic heart diseases	I24	7,767
Chronic ischemic heart disease	I25	346,684
Other pulmonary heart diseases	I27	25,148
Myocardiopathy	I42	16,000
Paroxysmal tachycardia	I47	6,315
Heart failure	I50	57,647
Heart disease complications	I51	11,198
Cerebral infarction	I63	339,629
Occlusion/stenosis of cerebral artery	I65	2,729
Other cerebrovascular diseases	I67	83,811

Summary statistics of air pollutants and metrological factors on the hospitalization dates are presented in Table 2. The averaged exposure levels of PM_10_ and PM_2.5_ were 82.7 μg/m^3^ (SD = 62.2 μg/m^3^) and 47.0 μg/m^3^ (SD = 39.3 μg/m^3^), respectively. The mean exposures of O_3_, NO_2_, SO_2_, and CO were 54.2, 38.5, 16.9, and 1.0 mg/m^3^, respectively. Temperature and relative humidity had a mean of 15.5 °C and 70.2%, respectively. The Spearman correlations among the air pollutants are summarized in Table S1. PM_2.5_ was strongly correlated with PM_10_ (*r*_*s*_ = 0.88), moderately correlated with NO_2_ (*r*_*s*_ = 0.54), SO_2_ (*r*_*s*_ = 0.50), and CO (*r*_*s*_ = 0.56), and negatively correlated with O_3_ (*r*_*s*_ = −0.13).

**Table 2:** Summary of the average concentration of air pollutants and metrological conditions on hospitalization dates.

Variables	Mean ± SD	Percentiles				
		Min	1-Q	Median	3-Q	Max
PM_10_ (μg/m^3^)	82.7 ± 62.2	15.2	45.5	69.1	105.3	314.0
PM_2.5_ (μg/m^3^)	47.0 ± 39.3	7.7	24.2	37.5	58.8	200.5
O_3_ (μg/m^3^)	54.2 ± 32.2	4.4	30.0	50.4	74.7	142.8
NO_2_ (μg/m^3^)	38.5 ± 21.0	5.9	24.0	36.0	50.0	103.0
SO_2_ (μg/m^3^)	16.9 ± 20.2	2.1	7.4	12.0	20.6	105.1
CO (mg/m^3^)	1.0 ± 0.6	0.2	0.7	0.9	1.3	3.4
Temperature (°C)	15.5 ± 10.5	−12.6	8.7	16.8	23.5	31.8
RH (%)	70.2 ± 18.0	23.0	59.0	73.0	83.0	98.0

SD, standard deviation; 1-Q, 25% percentiles; 3-Q, 75% percentiles; PM_10_, particulate matter with an aerodynamic diameter less than or equal to 10 μm; PM_2.5_, particulate matter with an aerodynamic diameter less than or equal to 2.5 μm; NO_2_, nitrogen dioxide; SO_2_, sulfur dioxide; O_3_, ozone; CO, carbon monoxide; RH, relative humidity.

**Fig. 1:**
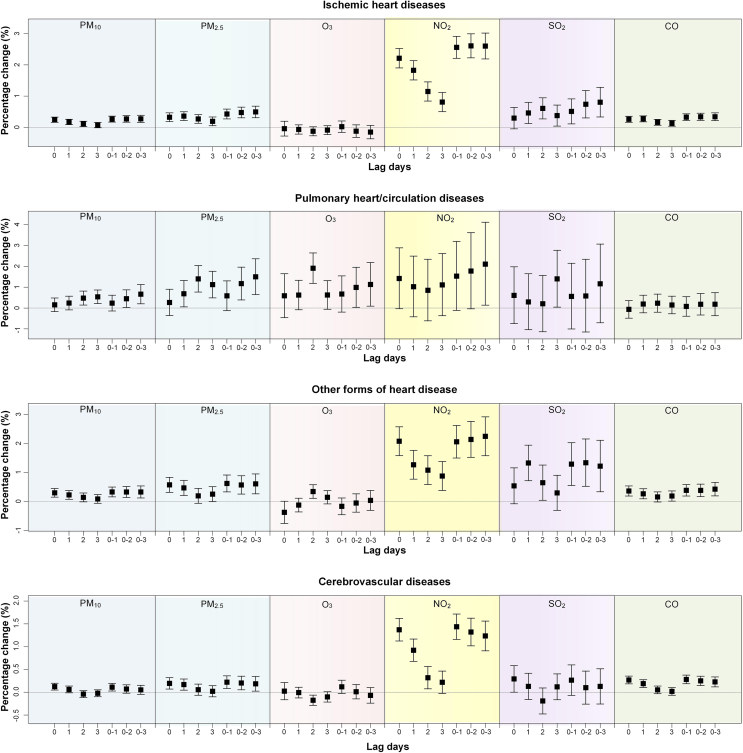
Percent changes in the hospitalization of major cardiovascular diseases associated with each 10 μg/m^3^ increase in air pollution concentrations (CO per 0.1 mg/m^3^) on different lag days. The boxes indicate the mean effect estimates, and the bars indicate the upper and lower 95% confidence intervals. Lag days, 0–1, the moving average of the current and previous day; 0–2, the moving average of the current and previous two days; 0–3, the moving average of the current and previous three days.

The percentage changes in hospitalization from four major categories of CVD associated with exposure to six air pollutants on different lag days are presented in Fig. 1. Although different endpoints exhibit distinct lag patterns, there were similar trends for a certain endpoint associated with all air pollutants. For example, the estimated associations were generally decreasing from lag 0 to lag 3 days, while we often observed the largest estimates with the narrowest confidence intervals on lag 0–1 day among moving average metrics. We thus chose lag 0–1 day as the main lag for the associations of all endpoints with each air pollutant.

**Fig. 2:**
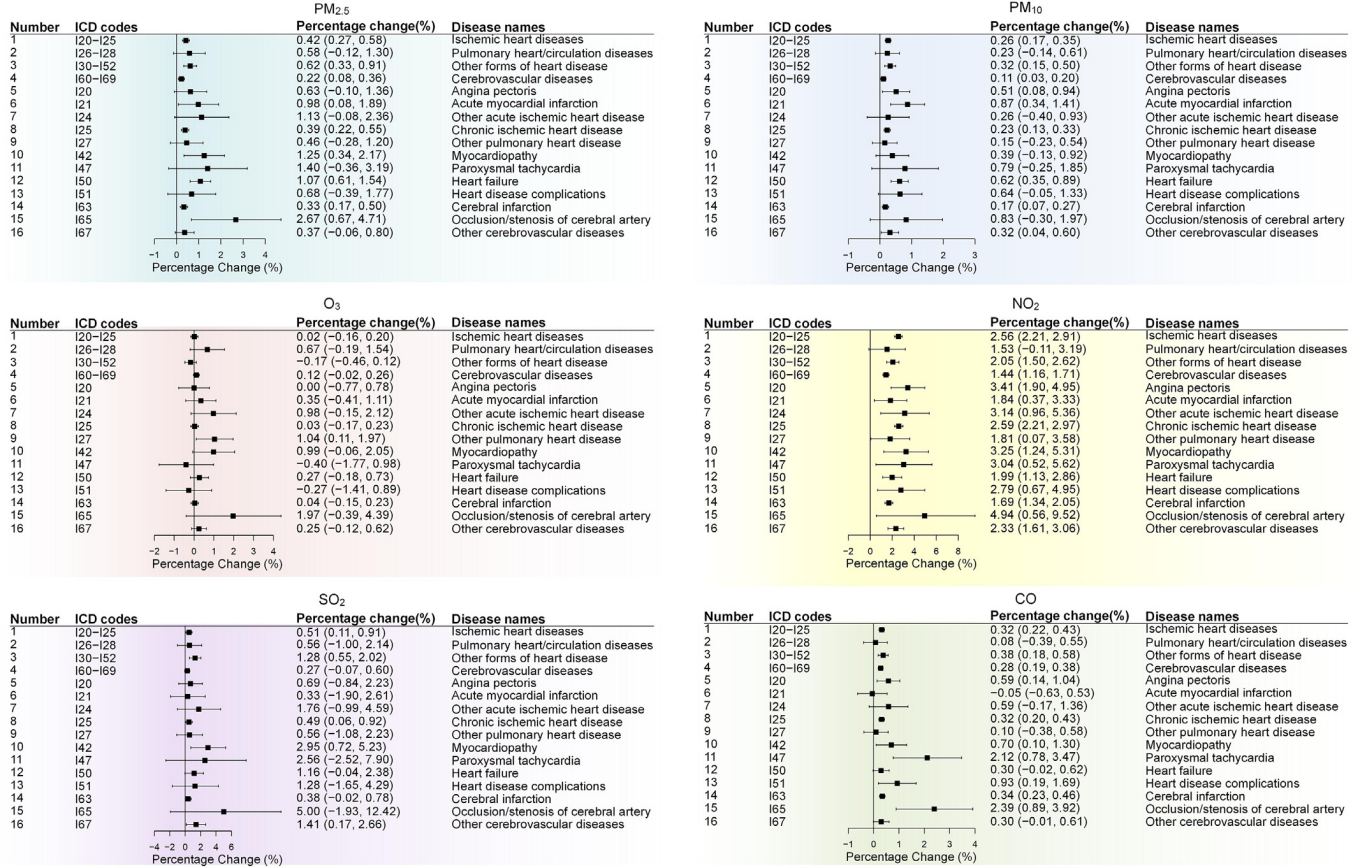
Percent changes in the hospitalization of cardiovascular diseases associated with air pollutant concentrations on lag 0–1 day. Results were presented as per 10 μg/m^3^ increase in air pollutant concentration, except for a 0.1 mg/m^3^ unit for CO. In the forest plots, the boxes indicate the mean effect estimates, and the bars indicate the upper and lower 95% confidence intervals.

The percentage changes in the hospitalization of all endpoints associated with per unit increase in air pollution concentrations on lag 0–1 day are illustrated in Fig. 2. Firstly, the two particles had similar effects on broad and specific CVD. For a 10 μg/m^3^ increase, it was found that both PM_2.5_ and PM_10_ were significantly associated with IHD, cerebrovascular diseases, and heart diseases of other forms. The corresponding percentage changes in hospitalizations were 0.42%, 0.22%, and 0.62% for PM_2.5_ and 0.26%, 0.11%, and 0.32% for PM_10_, respectively. For specific diseases, we observed positive and significant associations of acute myocardial infarction, chronic IHD, heart failure, and cerebral infarction with both PM_2.5_ and PM_10_. Secondly, for typical traffic-related air pollutants, NO_2_ and CO generally had positive and significant effects on the majority of CVD. In particular, NO_2_ was robustly associated with almost all cardiovascular endpoints, but pulmonary heart diseases and the effect estimates were consistently larger than those of other air pollutants. For a 10 μg/m^3^ increase in NO_2_, the associations for IHD, pulmonary heart/circulation diseases, other forms of heart disease, and cerebrovascular diseases were 2.56%, 1.53%, 2.05%, and 1.44%, respectively. Thirdly, for the remaining two gaseous air pollutants, the effect estimates for SO_2_ were all positive, but many of them did not reach statistical significance. However, for O_3_, no significant associations were observed for any endpoints, and the associations were leaning toward a negative direction. All *P* values were adjusted by false discovery rate (Table S2).

**Fig. 3:**
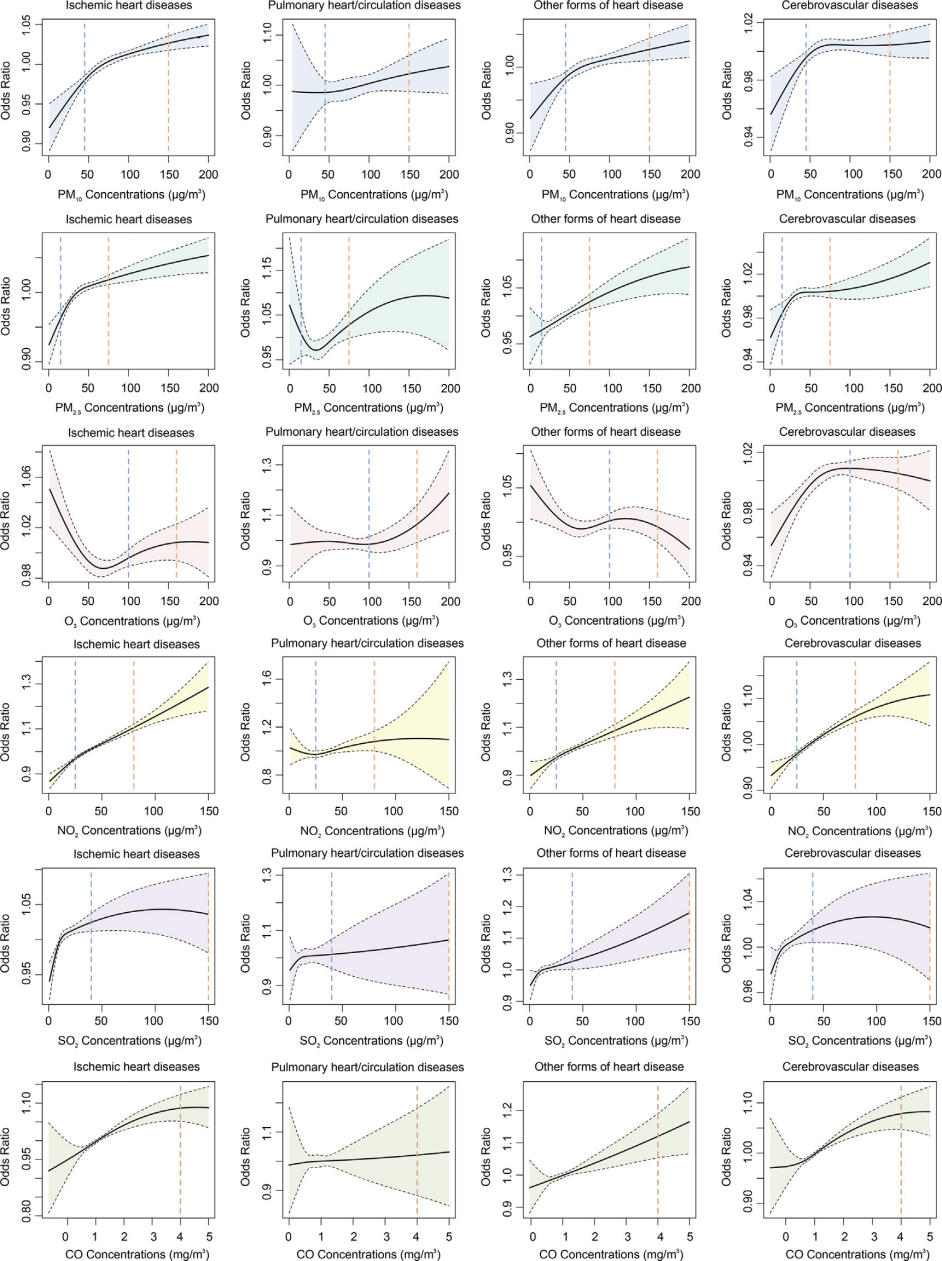
Concentration–response relationship curves of four major cardiovascular diseases associated with air pollution concentrations. The solid lines indicate the mean effect estimates, and the shades indicate the 95% confidence intervals. The dashed lines represent WHO (blue) and China (yellow) air quality regulation limits. WHO Air Quality Guidelines: PM_10_, 45 μg/m^3^; PM_2.5_, 15 μg/m^3^; O_3_, 100 μg/m^3^; NO_2_, 25 μg/m^3^; SO_2_, 40 μg/m^3^; CO, 4 mg/m^3^. China Air Quality Standard: PM_10_, 150 μg/m^3^; PM_2.5_, 75 μg/m^3^; O_3_, 160 μg/m^3^; NO_2_, 80 μg/m^3^; SO_2_, 150 μg/m^3^; CO, 4 mg/m^3^.

The concentration–response relationship curve for four major categories of CVD associated with six air pollutants is shown in Fig. 3. For PM_2.5_ and PM_10_, the curves were positive and increased for IHD, cerebrovascular diseases, and heart diseases of other forms, and there was a clear pattern that showed larger slopes with lower concentrations (<50 μg/m^3^). For NO_2_ and CO, the curves for the above three endpoints were almost linear and kept monotonically increased, while the wider confidence intervals at higher ranges might indicate less observations at such exposure ranges. The curves for SO_2_ and O_3_ were less fitted and converged, particularly for O_3_ with null associations. Notably, there were still significant associations of air pollutants below 2021 WHO Air Quality Guidelines and China Air Quality Standards (recommended 24-h levels) [28]. We visualized the concentration-response curves for twelve specific diseases with each air pollutant. Similarly, the curves for PM_2.5_, PM_10_, NO_2_, and CO fitted better than SO_2_ and O_3_, with mostly positive patterns across these endpoints (Fig. S2-S7).

The results of two pollutant models for the four categories of CVD are summarized in Table 3. To begin with, the estimates of PM_2.5_ and PM_10_ consistently decreased and even lost significance when mutually adjusted or with adjustment of NO_2_ and CO, but they did not change after controlling for SO_2_ and O_3_. Then, the associations of NO_2_ and CO were quite robust after adjusting for other air pollutants. Interestingly, NO_2_ was insensitive to the adjustment of any co-pollutants, including CO, but the association of CO would decrease when controlling for NO_2_. The associations of SO_2_ were only stable when controlling for O_3_, whereas the associations of O_3_ kept non-significant in both single and two pollutant models. In the sensitivity analysis with adjustment of atmospheric pressure, the overall associations between air pollutants and CVDs only slightly changed (Fig. S8).

**Table 3:** Associations between air pollutants and hospitalization of major cardiovascular diseases in single and two pollutant models.

Diseases	Models	PM_10_	PM_2.5_	O_3_	NO_2_	SO_2_	CO
Ischemic heart diseases	Unadjusted	0.26 (0.17, 0.35)	0.42 (0.27, 0.58)	0.02 (−0.16, 0.20)	2.56 (2.21, 2.91)	0.51 (0.11, 0.91)	0.32 (0.22, 0.43)
	+ PM_10_	NA	0.17 (−0.06, 0.40)	−0.04 (−0.22, 0.15)	2.64 (2.25, 3.04)	0.18 (−0.24, 0.59)	0.23 (0.12, 0.35)
	+ PM_2.5_	0.23 (0.08, 0.37)	NA	−0.05 (−0.23, 0.14)	2.92 (2.51, 3.34)	0.23 (−0.19, 0.66)	0.27 (0.14, 0.39)
	+ O_3_	0.25 (0.16, 0.35)	0.43 (0.27, 0.59)	NA	2.54 (2.19, 2.90)	0.48 (0.08, 0.88)	0.31 (0.20, 0.42)
	+ NO_2_	0.00 (−0.10, 0.10)	−0.07 (−0.24, 0.10)	−0.17 (−0.35, 0.02)	NA	−0.57 (−0.99, −0.14)	0.05 (−0.06, 0.17)
	+ SO_2_	0.26 (0.16, 0.35)	0.42 (0.26, 0.58)	0.00 (−0.18, 0.19)	2.78 (2.41, 3.15)	NA	0.33 (0.22, 0.44)
	+ CO	0.19 (0.09, 0.30)	0.30 (0.12, 0.48)	0.00 (−0.18, 0.18)	2.62 (2.23, 3.00)	0.14 (−0.29, 0.57)	NA
Pulmonary heart/circulation diseases	Unadjusted	0.23 (−0.14, 0.61)	0.58 (−0.12, 1.30)	0.67 (−0.19, 1.54)	1.53 (−0.11, 3.19)	0.56 (−1.00, 2.14)	0.08 (−0.39, 0.55)
	+ PM_10_	NA	0.69 (−0.29, 1.68)	0.68 (−0.19, 1.56)	1.43 (−0.32, 3.21)	0.34 (−1.25, 1.96)	−0.01 (−0.50, 0.49)
	+ PM_2.5_	0.16 (−0.37, 0.69)	NA	0.68 (−0.19, 1.56)	1.58 (−0.30, 3.50)	0.37 (−1.27, 2.04)	−0.01 (−0.55, 0.52)
	+ O_3_	0.23 (−0.15, 0.61)	0.56 (−0.15, 1.27)	NA	1.65 (−0.01, 3.34)	0.54 (−1.03, 2.13)	0.08 (−0.39, 0.55)
	+ NO_2_	0.13 (−0.26, 0.53)	0.35 (−0.42, 1.14)	0.65 (−0.22, 1.53)	NA	0.07 (−1.58, 1.75)	−0.10 (−0.61, 0.41)
	+ SO_2_	0.22 (−0.16, 0.60)	0.55 (−0.18, 1.28)	0.72 (−0.15, 1.59)	1.48 (−0.25, 3.24)	NA	0.04 (−0.45, 0.53)
	+ CO	0.27 (−0.12, 0.67)	0.74 (−0.05, 1.55)	0.74 (−0.13, 1.62)	1.90 (0.09, 3.75)	0.66 (−0.99, 2.34)	NA
Other forms of heart disease	Unadjusted	0.32 (0.15, 0.50)	0.62 (0.33, 0.91)	−0.17 (−0.46, 0.12)	2.05 (1.50, 2.62)	1.28 (0.55, 2.02)	0.38 (0.18, 0.58)
	+ PM_10_	NA	0.41 (−0.01, 0.84)	−0.24 (−0.53, 0.06)	1.97 (1.34, 2.60)	0.98 (0.22, 1.75)	0.27 (0.06, 0.49)
	+ PM_2.5_	0.11 (−0.15, 0.36)	NA	−0.27 (−0.57, 0.02)	1.95 (1.30, 2.61)	0.86 (0.08, 1.65)	0.23 (0.00, 0.46)
	+ O_3_	0.32 (0.15, 0.49)	0.64 (0.35, 0.93)	NA	2.00 (1.44, 2.56)	1.25 (0.52, 2.00)	0.37 (0.17, 0.57)
	+ NO_2_	0.06 (−0.13, 0.24)	0.13 (−0.19, 0.45)	−0.39 (−0.68, −0.10)	NA	0.25 (−0.53, 1.03)	0.10 (−0.12, 0.31)
	+ SO_2_	0.30 (0.13, 0.48)	0.59 (0.29, 0.89)	−0.18 (−0.47, 0.11)	2.13 (1.54, 2.73)	NA	0.37 (0.16, 0.58)
	+ CO	0.22 (0.03, 0.40)	0.43 (0.11, 0.76)	−0.19 (−0.48, 0.10)	1.90 (1.28, 2.52)	0.85 (0.06, 1.64)	NA
Cerebrovascular diseases	Unadjusted	0.11 (0.03, 0.20)	0.22 (0.08, 0.36)	0.12 (−0.02, 0.26)	1.44 (1.16, 1.71)	0.27 (−0.07, 0.60)	0.28 (0.19, 0.38)
	+ PM_10_	NA	0.13 (−0.08, 0.34)	0.09 (−0.06, 0.23)	1.57 (1.26, 1.88)	0.09 (−0.26, 0.44)	0.26 (0.16, 0.37)
	+ PM_2.5_	0.04 (−0.09, 0.16)	NA	0.08 (−0.07, 0.22)	1.65 (1.32, 1.97)	0.11 (−0.25, 0.46)	0.28 (0.17, 0.39)
	+ O_3_	0.11 (0.03, 0.19)	0.24 (0.10, 0.38)	NA	1.37 (1.09, 1.65)	0.24 (−0.10, 0.57)	0.28 (0.18, 0.37)
	+ NO_2_	−0.08 (−0.17, 0.01)	−0.12 (−0.27, 0.04)	−0.01 (−0.16, 0.13)	NA	−0.38 (−0.73, −0.03)	0.11 (0.01, 0.22)
	+ SO_2_	0.10 (0.02, 0.19)	0.20 (0.06, 0.34)	0.11 (−0.04, 0.25)	1.52 (1.22, 1.81)	NA	0.28 (0.18, 0.38)
	+ CO	0.01 (−0.08, 0.10)	0.02 (−0.14, 0.18)	0.10 (−0.05, 0.24)	1.29 (0.99, 1.59)	−0.13 (−0.49, 0.22)	NA

Notes: Estimates were presented as percentage changes (mean and 95% confidence intervals) associated with each 10 μg/m^3^ increase in air pollutant concentrations (CO per 0.1 mg/m^3^).

## 4 Discussion

In this national case-crossover study, we found that short-term exposure to ambient PM_2.5_, PM_10_, NO_2_, and CO was significantly associated with increased hospitalization of IHD, cerebrovascular diseases, and heart diseases of other forms. Among these broad categories of CVD, specific causes, such as acute myocardial infarction, myocardiopathy, heart failure, cerebral infarction, and occlusion of the cerebral artery, were closely linked to exposure to air pollutants. The concentration–response relationships between CVDs and most air pollutants (except for O_3_) were positive and linear, and the associations remained even below the most stringent 24-h levels of the 2021 WHO Air Quality Guidelines for ambient air pollutants. The associations of NO_2_ were the most robust in two pollutant models. The results of this analysis provided evidence to understand the health effects of ambient air pollution on specific causes of CVDs and may aid targeted controlling strategy against environmental risk factors for cardiovascular inpatients.

Numerous epidemiological studies demonstrated the link between PM and CVDs morbidity. Our results showed that a 10 μg/m^3^ increase in PM_2.5_ and PM_10_ was associated with an increment in the hospitalization of IHD and cerebrovascular disease, and the corresponding increases were 0.42% and 0.22% for PM_2.5_ and 0.26% and 0.11% for PM_10_. The magnitude of effects was comparable to previous multicity studies on PM and CVDs. For example, Tian et al. [29] examined the associations between PM_2.5_ and hospital admission for cause-specific CVD in 184 Chinese cities and reported an increase of 0.26% for total CVDs, 0.31% for IHD, and 0.27% for heart failure. Dominici et al. [30] analyzed the hospital admission time-series data in 204 US counties and found significant effects of PM_2.5_ that increased 0.80% cerebrovascular disease and 0.25% IHD. Similar results have been reported in other single-city studies. For a 10 μg/m^3^ increase, Xie et al. [31] reported a 0.27% increase in IHD hospitalization associated with PM_2.5_ in Beijing; Oudin et al. [32] found significant effects of PM_10_ on 13% increase in ischemic stroke hospital admission in Sweden. Lokotola et al. [33] observed a 6.3% increase in CVD hospitalization associated with PM_10_ in Cape Town. Overall, our results reinforced the established associations between PM and CVD morbidity in China.

Typical traffic-related air pollutants, such as NO_2_ and CO, have been widely reported to be associated with CVD morbidity in previous studies, and the effect estimates were generally larger than those of PM [[8], [9], [10],^12^,^34^]. For instance, Tian et al. [8] conducted a nationwide time-series study in 218 Chinese cities and found that a 10 μg/m^3^ in NO_2_ and a 0.1 mg/m^3^ increase in CO; the corresponding increase in total hospital admission was 1.68% and 0.26%, respectively. Barnett et al. [12] conducted a case-crossover study in seven cities in Australia and New Zealand and found that a 3.0% increase in total CVD and a 2.5% increase in IHD hospital admission were associated with NO_2_, while the estimates for a unit increase in CO were 2.2% for total CVD and 2.3% for IHD. Vidale et al. [35] reported an increment of 3.9% in stroke admission associated with NO_2_ in Italy. In our study, a 10 μg/m^3^ increase in NO_2_ was associated with a 2.57% increase in hospitalization of IHD and 2.07% in cerebrovascular diseases, while the estimates for a 0.1 mg/m^3^ increase in CO were 0.26% for IHD and 0.27% for cerebrovascular diseases. The larger estimates of NO_2_ and CO on CVD were interesting to think of, and whether this indicated a combined effect of other traffic-related factors, such as noise, remained to be further discovered.

The effect of SO_2_ on CVD has not been systematically studied, and a lack of relevant evidence may have led to the loosened recommended level of 40 μg/m^3^ for 24-h SO_2_ in the new version of WHO Air Quality Guidelines [28] from the previous 20 μg/m^3^ daily level [36]. There may emerge a motive to reassess the short-term associations of SO_2_ with mortality or morbidity outcomes. We found that a 10 μg/m^3^ increase in SO_2_ was significantly associated with a 0.51% increase in IHD hospitalization. The corresponding estimates for SO_2_ and total hospital admission in the national study by Tian et al. were 1.16% [8]. Vidale et al. [35] reported an increment of 3.1% in stroke admission associated with each 10 μg/m^3^ increase in SO_2_ in Italy. More relevant studies are needed for a comprehensive evaluation of the health impact of SO_2_.

There has always been a debate on whether O_3_ has a short-term health impact on CVDs. In previous studies of O_3_, researchers have mostly reported negative or non-significant findings. For example, in the Italian study by Vidale et al. [35], PM and other gaseous air pollutants significantly increased the admission rate of stroke, while O_3_ constantly showed negative effects. The case-crossover study in seven cities in Australia and New Zealand also reported that all pollutants were significantly associated with five categories of CVD admissions, except for O_3_ [12]. Our study generally found negative but not significant associations of O_3_. This phenomenon might be explained by the hypothesis that short-term exposure to O_3_ may cause vasodilation in the circulation system, and low levels of O_3_ have been clinically used as a sort of treatment for CVD [37]. Nevertheless, the exact associations between O_3_ and CVD need further verification.

Despite the abundance of epidemiological evidence, the underlying mechanism in the observed associations between air pollutants and CVDs need to be further interpreted. The direct effects of pollutants on the cardiovascular system, blood, and lung receptors and/or the indirect effects due to pulmonary oxidative stress and inflammatory responses have been proposed [19,38]. The direct effects of PM could induce acute cardiovascular responses, as it can breach the pulmonary alveoli and enter the circulation system. The indirect effects could reflect the contribution of gaseous air pollutants that induce systemic inflammatory states, such as impaired vascular function and atherosclerosis progression. The above process has been verified by *in vitro* and *in vivo* studies [39,40]. Some new pathways, such as epigenetic changes, reactive oxygen species (ROS) generation [41], activation of the hypothalamic–pituitary–adrenal (HPA) axis [42], and disturbance in the autonomic nervous system [43], were recently hypothesized to play a potential role. For this analysis, all air pollutants except for O_3_ showed consistently positive associations with CVDs. Exposure to O_3_ has been closely linked with respiratory mortality and morbidity [44,45], but its effect on CVDs has always been mixed, especially for short-term exposures. The current study may also provide insights for future toxicological studies by extensively explore how air pollution exposure causes cardiovascular impact.

The current study provided several references for future research. First, the case-crossover study design using individual cases simultaneously avoids potential confounding from time-invariant factors, offering greater evidence strength than ecological time-series studies. Second, we obtained data from a large national hospital-based registry with exact diagnosis information for hospitalization events, which enables us to conduct exploratory analyses on a wide spectrum of CVDs that are associated with air pollution, thus avoiding potential publication bias. Third, we systematically assessed the effects of all criteria air pollutants on CVD hospitalizations in China with wide exposure ranges. Furthermore, we found significant associations even under the most stringent regulations, hence, providing scientific support for continuing air pollution control.

This study was subject to some limitations. First, the analysis was based on measurements from ground monitors rather than high-resolution modeling, and exposure assignment was realized at the hospital level instead of residential addresses. Therefore, exposure measurement error may be inevitable. Furthermore, a larger exposure bias would be introduced for participants living far away from the hospitals. However, as pointed out by previous studies, such non-differential misclassification could only cause Berkson bias and would not influence the central estimates of the associations but only the inflation of confidence intervals [46]. Second, limited by statistical power, this analysis failed to examine the effects of air pollution on specific CVDs with very low incidence rates. Furthermore, although stringent quality control procedure was adopted for disease diagnosis, the likelihood of outcome misclassification by ICD coding errors is inevitable, which may weaken the evidence for the outcomes. Third, we did not have information on medication use or compliance for each patient. However, as medication use would not influence the air pollution levels of participants, it is not a confounder in the associations between air pollution exposure and CVD hospitalization, but it may be a potential effect modifier. Lastly, we did not have enough information on location- or region-specific characteristics, which limited our ability to further quantify the regional heterogeneities.

## 5 Conclusion

Overall, we found significant associations between short-term exposure to ambient air pollution and increased hospitalization of a wide spectrum of CVDs in this nationwide study. These endpoints included IHD, cerebrovascular disease, heart diseases of other forms, and multiple specific CVDs. NO_2_ had the most robust effect among all air pollutants. The concentration-response curves for most air pollutants (except for O_3_) were positive and linear, and there were significant associations even below the daily regulation levels recommended by WHO Air Quality Guidelines and China Air Quality Standards. This study provided comprehensive evidence on the health effects of ambient air pollution on a wide spectrum of cardiovascular morbidity, striking an alert for potential CVD patients against these environmental risk factors.

## Author contributions

H.K. and J.X. are the joint corresponding authors and contributed equally to the conceptualization, funding acquisition, project administration, and supervision. C.L. and R.C. are the joint first authors and contributed equally to data curation, investigation, methodology, formal analysis, and writing-original draft. X.M. and W.W. contributed to methodology, software, and validation. J.L., Y.Z., and L.Z. contributed to data curation and investigation. All authors contributed to writing–review & editing.

## Declaration of competing interest

The authors declare they have no actual or potential competing financial interests.
