# Supplementary material for: Criteria air pollutants and hospitalizations of a wide spectrum of cardiovascular diseases: A nationwide case-crossover study in China
Source: Eco Environ Health. 2022 Nov 17;1(4):204–11. doi: 10.1016/j.eehl.2022.10.002 (PMC10702887; doi:10.1016/j.eehl.2022.10.002)
Supplement: Multimedia component 1 [file mmc1.docx]

**Supplemental information**

**Criteria air pollutants and hospitalizations of a wide spectrum of cardiovascular diseases: a nationwide case-crossover study in China**

Cong Liu^1, †^, Renjie Chen^1, †^, Xia Meng^1^, Weidong Wang^1^, Jian Lei^1^, Yixiang Zhu^1^, Lu Zhou^1^, Haidong Kan^1, 2, 3, *^, Jianwei Xuan^4,^ ^*^

^1^ School of Public Health, Shanghai Institute of Infectious Disease and Biosecurity, Key Lab of Public Health Safety of the Ministry of Education and NHC Key Lab of Health Technology Assessment, Fudan University, Shanghai 200032, China;

^2^ Children’s Hospital of Fudan University, National Center for Children’s Health, Shanghai 201102, China;

^3^ Shanghai Institute of Pollution Control and Ecological Security, Shanghai 200092, China;

^4^ Health Economic Research Institute, School of Pharmacy, Sun Yat-Shen University, Guangzhou 510275, China.

^†^ These authors contributed equally to this work.

***Corredponding authors**: [kanh@fudan.edu.cn](mailto:kanh@fudan.edu.cn) (HD Kan); [xuanjw3@sysu.edu.cn](mailto:xuanjw3@sysu.edu.cn) (JW Xuan).

# Supplementary methods

A time-stratified case-crossover study design was adopted to evaluate the association between short-term exposure to six criteria air pollutants and hospitalizations of cardiovascular diseases. Conditional logistic regression models were applied to investigate the associations. Temperature and relative humidity were adjusted according to previous studies. We used Cox regression models to realize conditional logistic regression. The formula can be described as follows:

$$LogHR\left( Y-time, event \right)\sim Pollutant+ns(temp,df)+ns(rh,df)+strata(id)$$

where *time* is the survival duration, *event* is the indicator for case or control, *Pollutant* is the air pollutant of interest, *temp* is temperature, *rh* is relative humidity, *df* is the degree of freedom, *id* is the identity coding for each individual.

We performed the statistical analyses using R software (Version 3.6.1) with the “survival” package. The core R code is described as follows:

*model* < -*coxph(Surv(time,event)~pollutant + ns(l03temp,3) + ns(l03rh,3) + strata(studyid), data = data, na.action = na.exclude)*

# Table S1. Spearman correlation coefficients among the air pollutants.

| Pollutants | PM_2.5_ | PM_10_ | NO_2_ | SO_2_ | O_3_ |
| --- | --- | --- | --- | --- | --- |
| PM_10_ | 0.88 |  |  |  |  |
| NO_2_ | 0.54 | 0.50 |  |  |  |
| SO_2_ | 0.50 | 0.50 | 0.38 |  |  |
| O_3_ | -0.13 | -0.04 | -0.35 | -0.09 |  |
| CO | 0.56 | 0.49 | 0.48 | 0.43 | -0.34 |

PM_10_, particulate matter with an aerodynamic diameter less than or equal to 10 μm; PM_2.5_, particulate matter with an aerodynamic diameter less than or equal to 2.5 μm; NO_2_, nitrogen dioxide; SO_2_, sulfur dioxide; O_3_, ozone; CO, carbon monoxide.

# Table S2. Associations between air pollutants and hospitalization of cardiovascular diseases with false detective rate adjusted P values.

| Endpoints | PM_2.5_ | | PM_10_ | | O_3_ | | NO_2_ | | SO_2_ | | CO | |
| --- | --- | --- | --- | --- | --- | --- | --- | --- | --- | --- | --- | --- |
|  | Estimate | P_FDR_ | Estimate | P_FDR_ | Estimate | P_FDR_ | Estimate | P_FDR_ | Estimate | P_FDR_ | Estimate | P_FDR_ |
| Ischemic heart diseases | 0.42 (0.27, 0.58) | <0.001 | 0.26 (0.17, 0.35) | <0.001 | 0.02 (-0.16, 0.20) | 1.000 | 2.56 (2.21, 2.91) | <0.001 | 0.51 (0.11, 0.91) | 0.069 | 0.32 (0.22, 0.43) | <0.001 |
| Pulmonary heart/circulation diseases | 0.58 (-0.12, 1.30) | 0.131 | 0.23 (-0.14, 0.61) | 0.255 | 0.67 (-0.19, 1.54) | 0.346 | 1.53 (-0.11, 3.19) | 0.070 | 0.56 (-1.00, 2.14) | 0.552 | 0.08 (-0.39, 0.55) | 0.810 |
| Other forms of heart disease | 0.62 (0.33, 0.91) | <0.001 | 0.32 (0.15, 0.50) | 0.001 | -0.17 (-0.46, 0.12) | 1.000 | 2.05 (1.50, 2.62) | <0.001 | 1.28 (0.55, 2.02) | 0.011 | 0.38 (0.18, 0.58) | 0.001 |
| Cerebrovascular diseases | 0.22 (0.08, 0.36) | 0.004 | 0.11 (0.03, 0.20) | 0.019 | 0.12 (-0.02, 0.26) | 0.338 | 1.44 (1.16, 1.71) | <0.001 | 0.27 (-0.07, 0.60) | 0.237 | 0.28 (0.19, 0.38) | <0.001 |
| Angina pectoris | 0.63 (-0.10, 1.36) | 0.126 | 0.51 (0.08, 0.94) | 0.039 | 0.00 (-0.77, 0.78) | 1.000 | 3.41 (1.90, 4.95) | <0.001 | 0.69 (-0.84, 2.23) | 0.501 | 0.59 (0.14, 1.04) | 0.020 |
| Acute myocardial infarction | 0.98 (0.08, 1.89) | 0.060 | 0.87 (0.34, 1.41) | 0.004 | 0.35 (-0.41, 1.11) | 0.675 | 1.84 (0.37, 3.33) | 0.019 | 0.33 (-1.90, 2.61) | 0.786 | -0.05 (-0.63, 0.53) | 0.875 |
| Other acute ischemic heart disease | 1.13 (-0.08, 2.36) | 0.111 | 0.26 (-0.40, 0.93) | 0.452 | 0.98 (-0.15, 2.12) | 0.338 | 3.14 (0.96, 5.36) | 0.009 | 1.76 (-0.99, 4.59) | 0.350 | 0.59 (-0.17, 1.36) | 0.160 |
| Chronic ischemic heart disease | 0.39 (0.22, 0.55) | <0.001 | 0.23 (0.13, 0.33) | <0.001 | 0.03 (-0.17, 0.23) | 1.000 | 2.59 (2.21, 2.97) | <0.001 | 0.49 (0.06, 0.92) | 0.085 | 0.32 (0.20, 0.43) | <0.001 |
| Other pulmonary heart disease | 0.46 (-0.28, 1.20) | 0.225 | 0.15 (-0.23, 0.54) | 0.452 | 1.04 (0.11, 1.97) | 0.338 | 1.81 (0.07, 3.58) | 0.046 | 0.56 (-1.08, 2.23) | 0.552 | 0.10 (-0.38, 0.58) | 0.804 |
| Myocardiopathy | 1.25 (0.34, 2.17) | 0.017 | 0.39 (-0.13, 0.92) | 0.187 | 0.99 (-0.06, 2.05) | 0.338 | 3.25 (1.24, 5.31) | 0.003 | 2.95 (0.72, 5.23) | 0.069 | 0.70 (0.10, 1.30) | 0.035 |
| Paroxysmal tachycardia | 1.40 (-0.36, 3.19) | 0.139 | 0.79 (-0.25, 1.85) | 0.187 | -0.40 (-1.77, 0.98) | 1.000 | 3.04 (0.52, 5.62) | 0.024 | 2.56 (-2.52, 7.90) | 0.495 | 2.12 (0.78, 3.47) | 0.005 |
| Heart failure | 1.07 (0.61, 1.54) | <0.001 | 0.62 (0.35, 0.89) | <0.001 | 0.27 (-0.18, 0.73) | 0.489 | 1.99 (1.13, 2.86) | <0.001 | 1.16 (-0.04, 2.38) | 0.143 | 0.30 (-0.02, 0.62) | 0.092 |
| Heart disease complications | 0.68 (-0.39, 1.77) | 0.225 | 0.64 (-0.05, 1.33) | 0.113 | -0.27 (-1.41, 0.89) | 1.000 | 2.79 (0.67, 4.95) | 0.015 | 1.28 (-1.65, 4.29) | 0.501 | 0.93 (0.19, 1.69) | 0.026 |
| Cerebral infarction | 0.33 (0.17, 0.50) | <0.001 | 0.17 (0.07, 0.27) | 0.003 | 0.04 (-0.15, 0.23) | 1.000 | 1.69 (1.34, 2.05) | <0.001 | 0.38 (-0.02, 0.78) | 0.143 | 0.34 (0.23, 0.46) | <0.001 |
| Occlusion/stenosis of cerebral artery | 2.67 (0.67, 4.71) | 0.019 | 0.83 (-0.30, 1.97) | 0.190 | 1.97 (-0.39, 4.39) | 0.338 | 4.94 (0.56, 9.52) | 0.035 | 5.00 (-1.93, 12.42) | 0.307 | 2.39 (0.89, 3.92) | 0.005 |
| Other cerebrovascular diseases | 0.37 (-0.06, 0.80) | 0.126 | 0.32 (0.04, 0.60) | 0.044 | 0.25 (-0.12, 0.62) | 0.436 | 2.33 (1.61, 3.06) | <0.001 | 1.41 (0.17, 2.66) | 0.085 | 0.30 (-0.01, 0.61) | 0.082 |


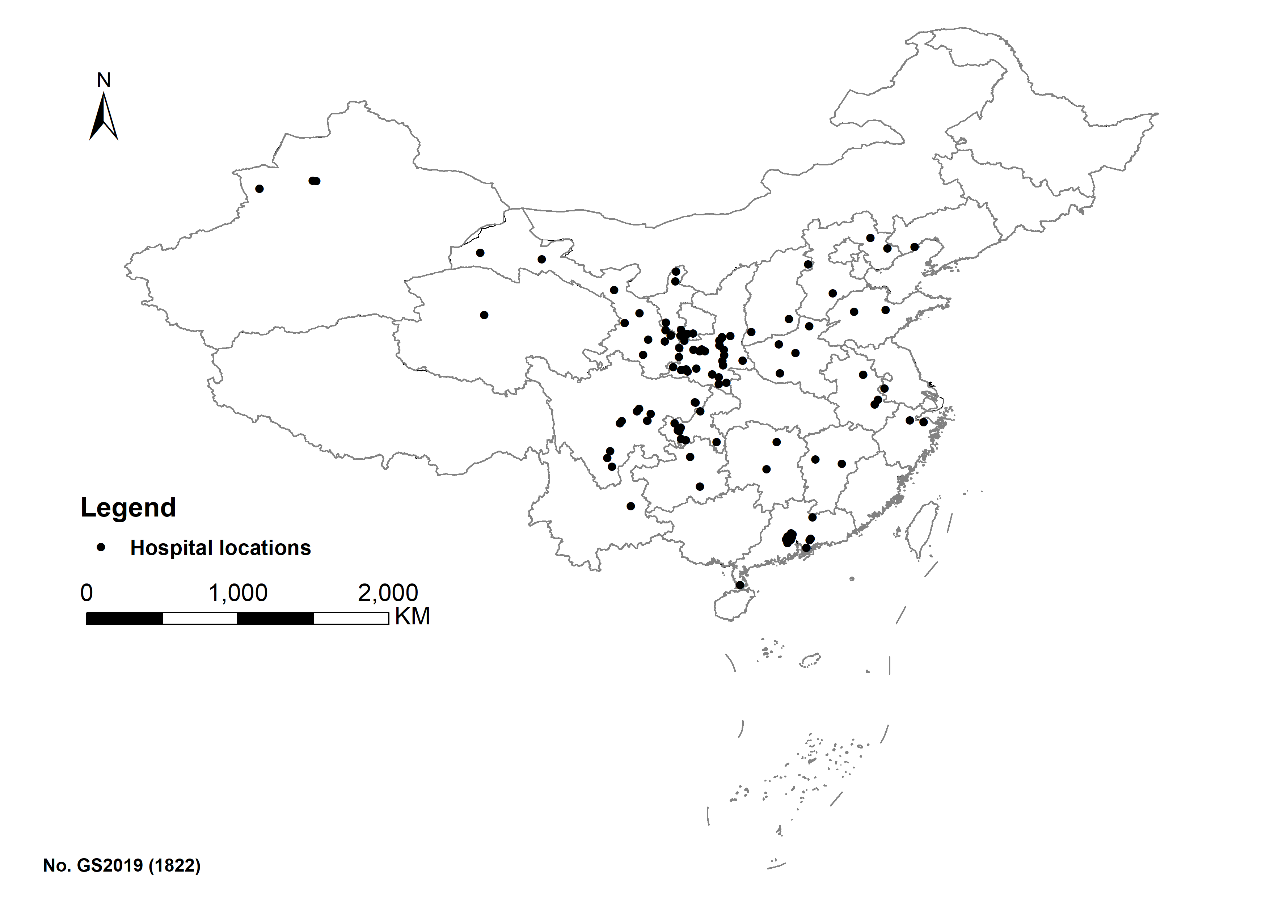


# Figure S1. Locations of hospitals included in this study


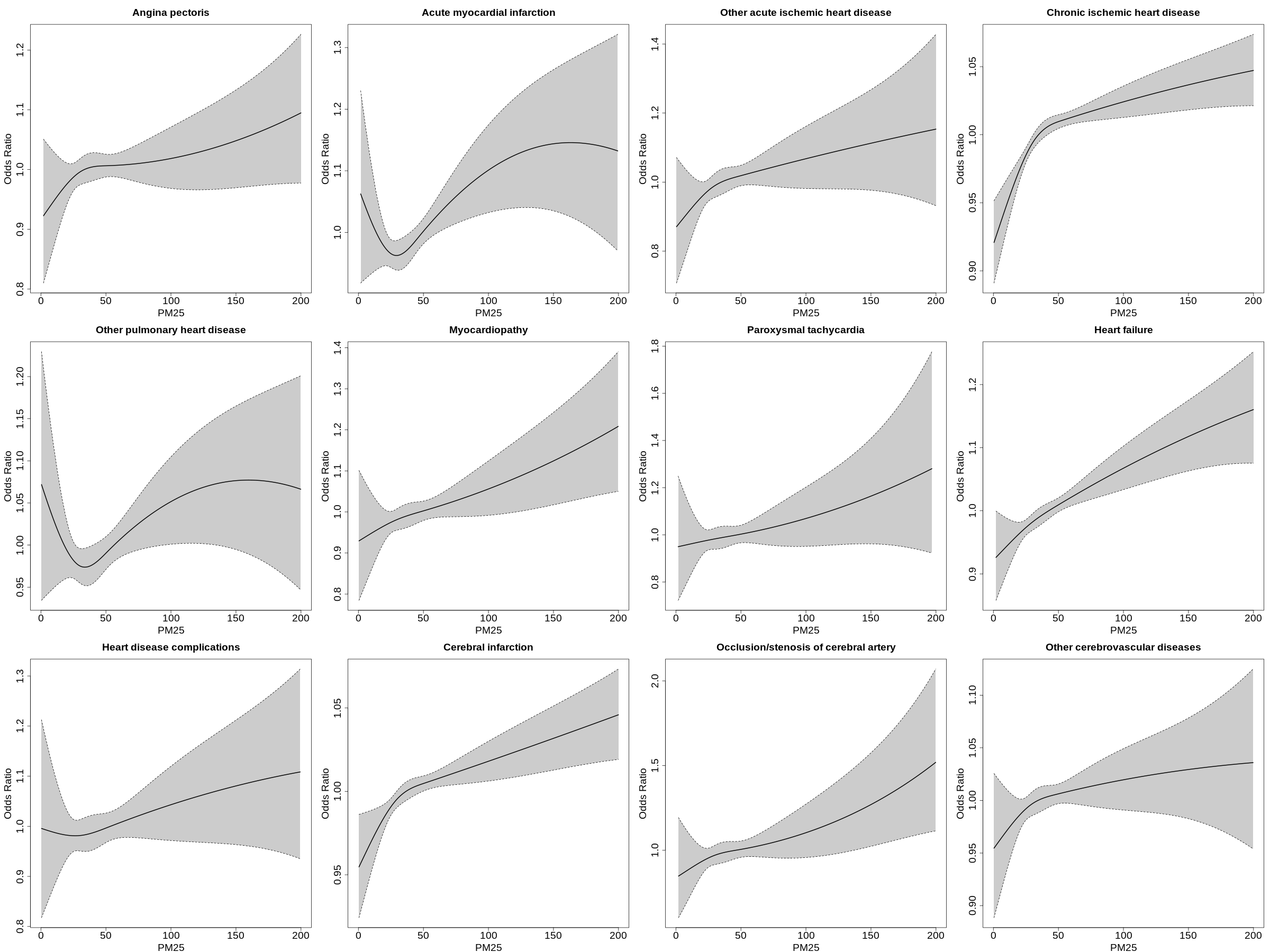


Figure S2. Concentration-response relationship curves of 12 specific cardiovascular diseases associated with PM_2.5_ on lag 0-1 day


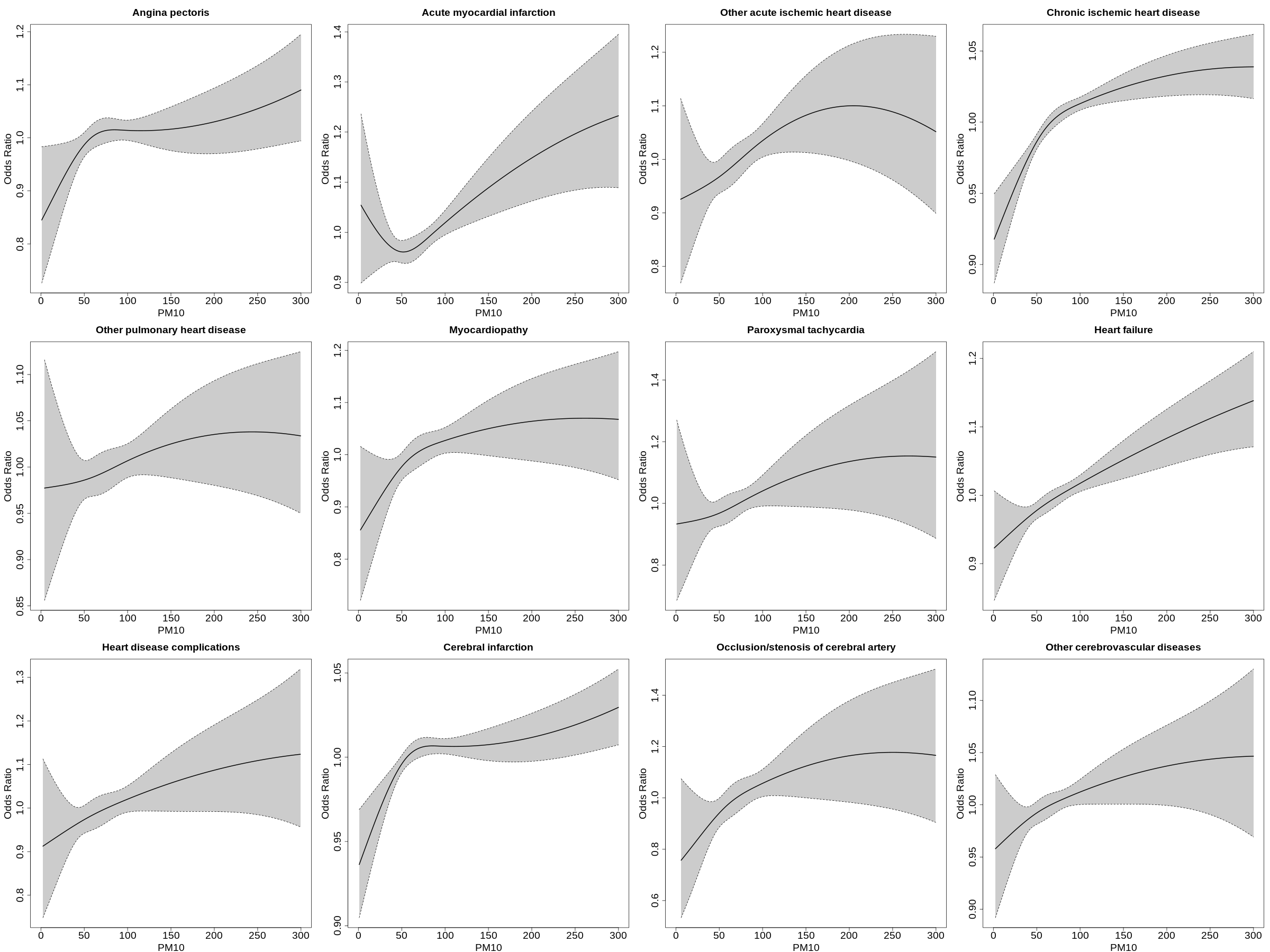


Figure S3. Concentration-response relationship curves of 12 specific cardiovascular diseases associated with PM_10_ on lag 0-1 day


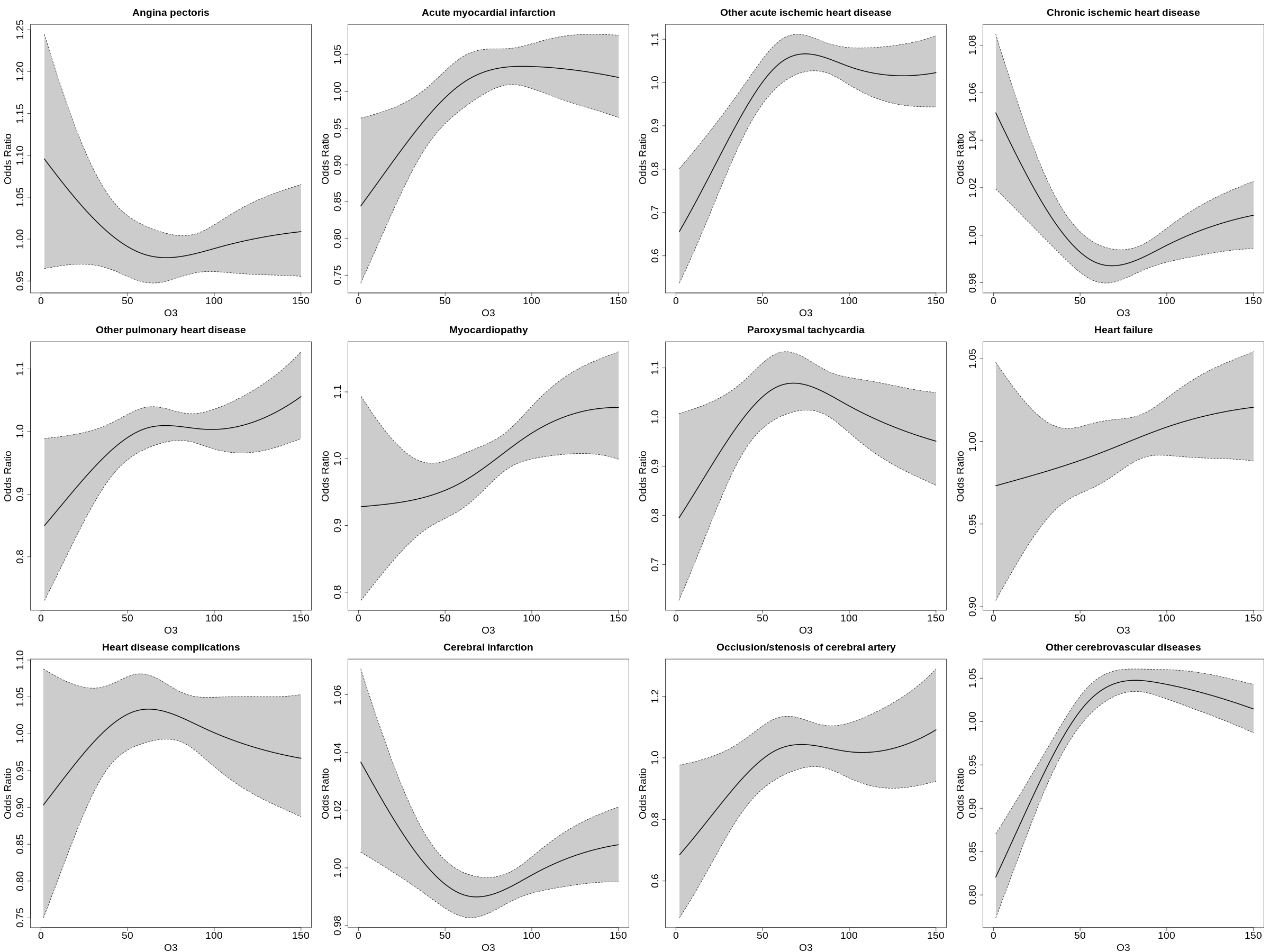


Figure S4. Concentration-response relationship curves of 12 specific cardiovascular diseases associated with O_3_ on lag 0-1 day


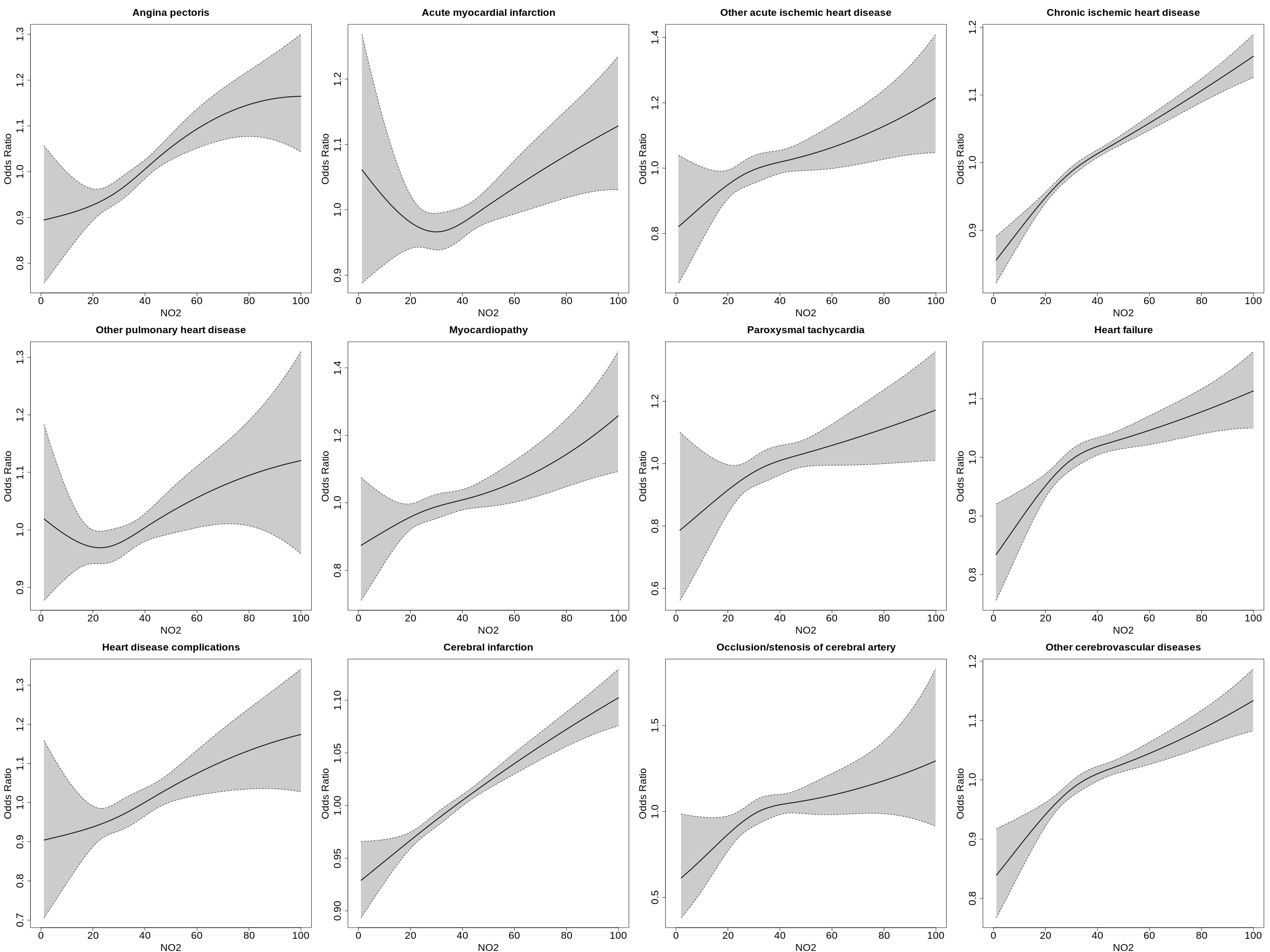


Figure S5. Concentration-response relationship curves of 12 specific cardiovascular diseases associated with NO_2_ on lag 0-1 day


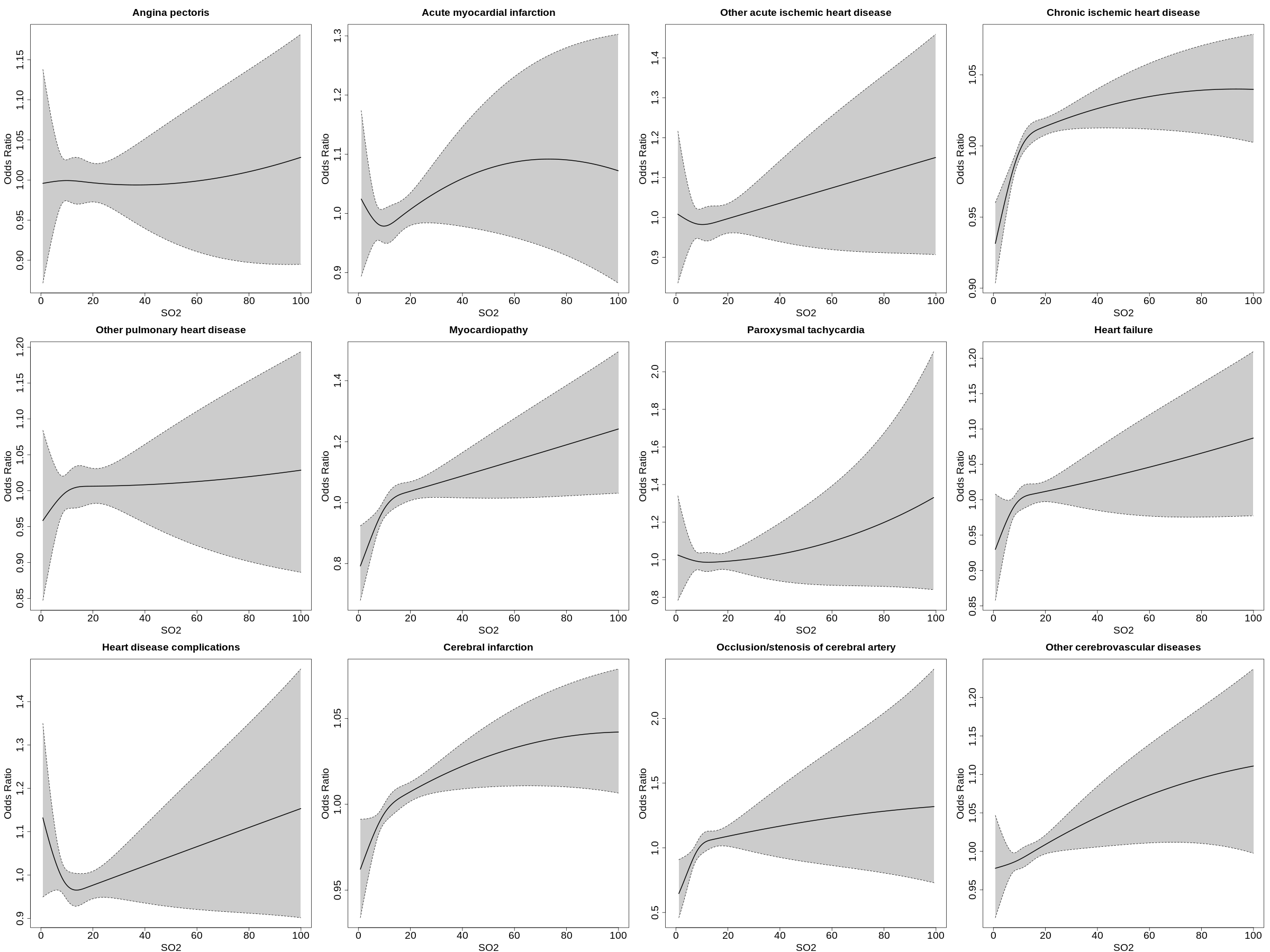


Figure S6. Concentration-response relationship curves of 12 specific cardiovascular diseases associated with SO_2_ on lag 0-1 day


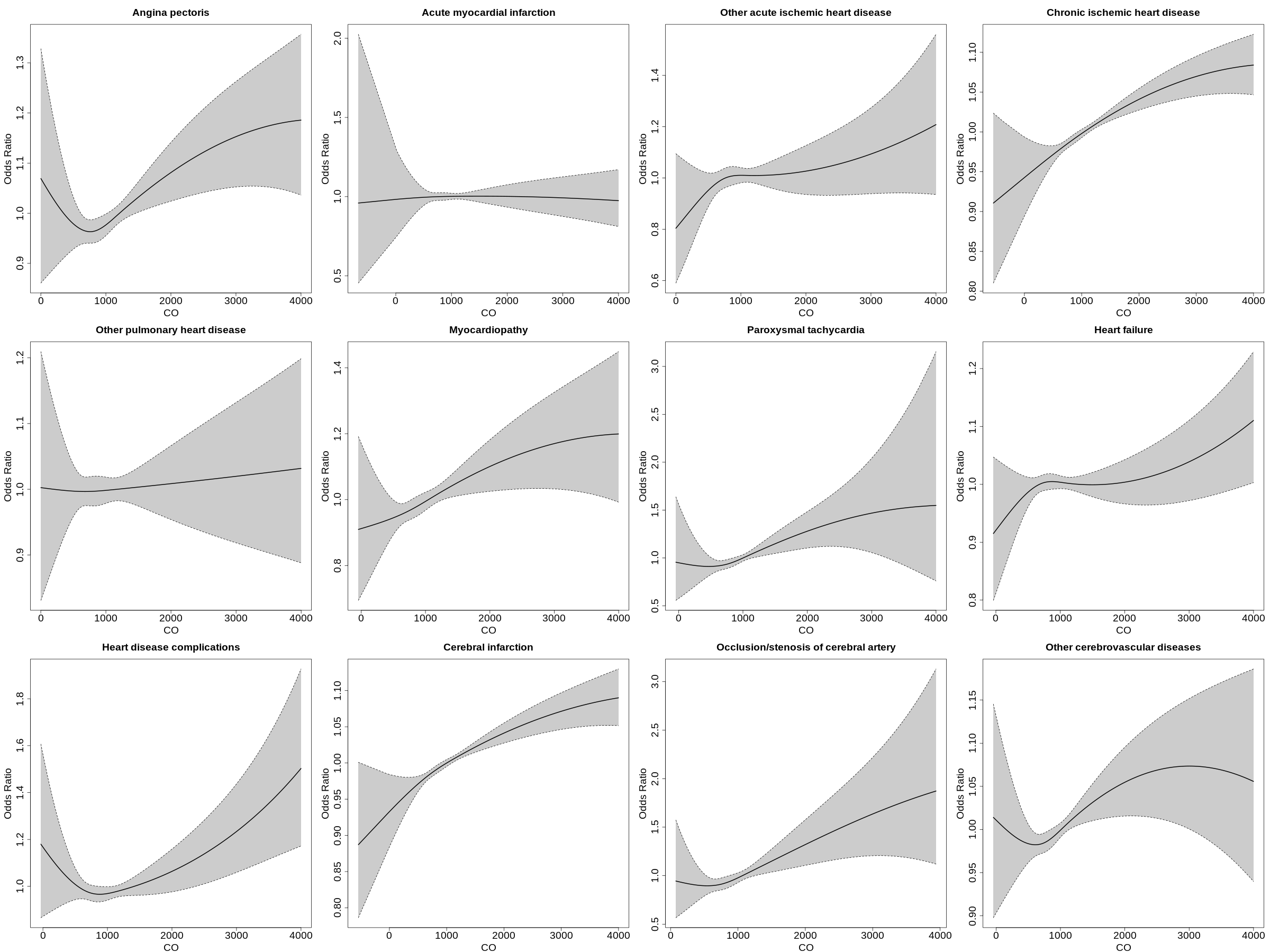


Figure S7. Concentration-response relationship curves of 12 specific cardiovascular diseases associated with CO on lag 0-1 day


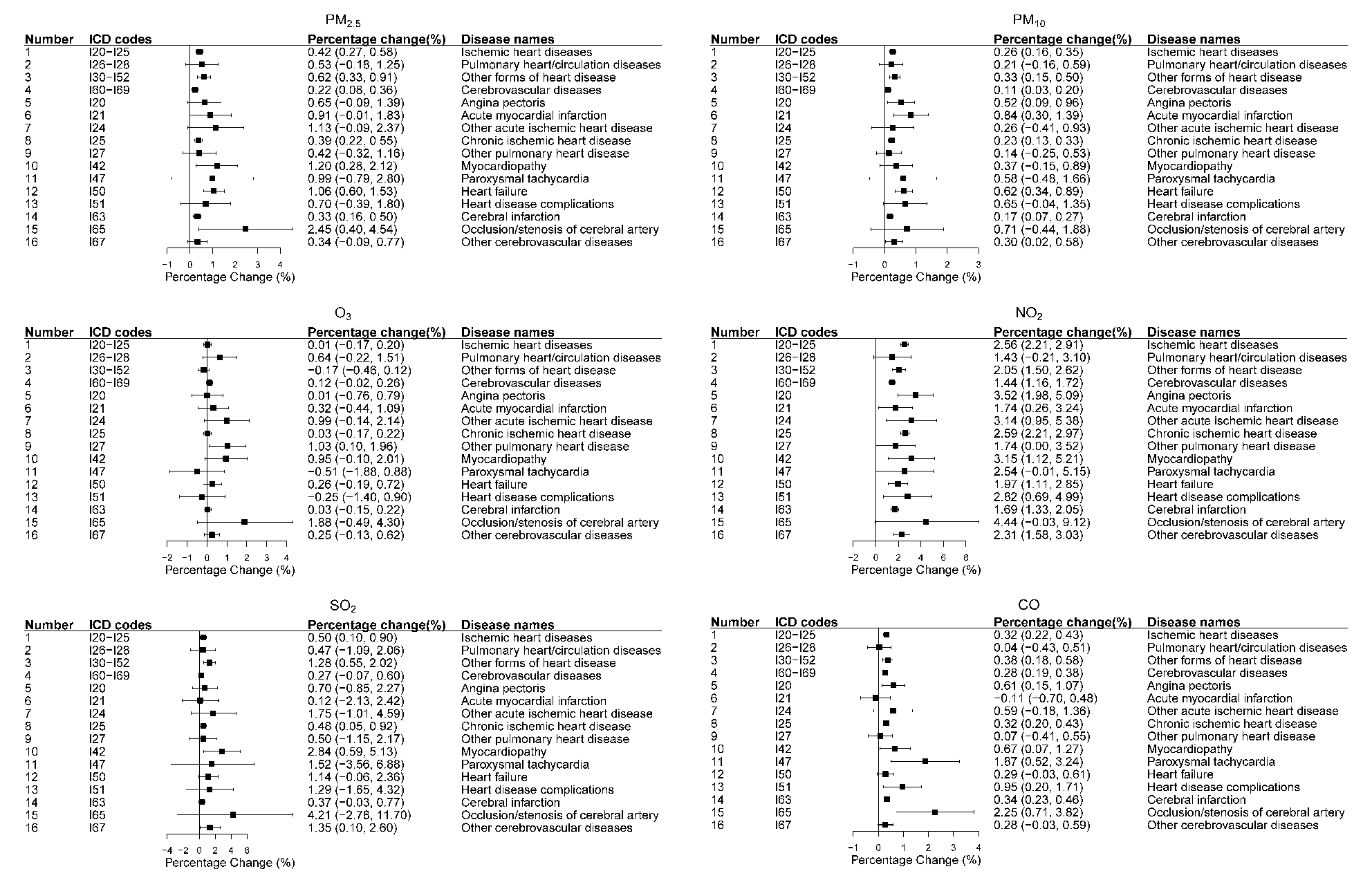


# Figure S8. Percent changes in hospitalization of cardiovascular diseases associated with air pollutant concentrations on lag 0-1 day with adjustment of atmospheric pressure. Results were presented as per 10 μg/m^3^ increase in air pollutant concentration, except for a 0.1mg/m^3^ unit for CO. In the forest plots, the boxes indicate the mean effect estimates, and the bars indicate the upper and lower 95% confidence intervals.
